# Editorial: Frontiers in cancer neuroscience: unraveling the complex interplay of neural mechanisms in tumorigenesis and therapeutics

**DOI:** 10.3389/fonc.2026.1980480

**Published:** 2026-09-28

**Authors:** Josh Neman, Daniel A. Pomeranz Krummel, Soma Sengupta

**Affiliations:** 1 Department of Neurological Surgery, Physiology & Neuroscience, USC Brain Tumor Center, Norris Comprehensive Cancer Center, Keck School of Medicine, University of Southern California, Los Angeles, CA, United States; 2 Department of Neurosurgery, Tufts Medical Center, Boston, MA, United States; 3 Department of Neurology, Tufts Medical Center, Boston, MA, United States

**Keywords:** cancer neuroscience, glioma, neural tumor interactions, neuro-immune signaling, tumor microenvironment

Over the past decade, discoveries demonstrating reciprocal communication between neurons, glial cells, immune cells, and cancer have significantly impacted our understanding of tumor biology, revealing that the nervous system is not only a target of malignancy but an active regulator of tumor initiation, progression, metastasis, therapeutic response, and disease recurrence (1). Importantly, this has revealed potential opportunities for developing therapies that target neural–tumor interactions.

The Research Topic, *Frontiers in Cancer Neuroscience: Unraveling the Complex Interplay of Neural Mechanisms in Tumorigenesis and Therapeutics*, captures the breadth of this rapidly expanding field in its ten articles that include five reviews, two mini-reviews, two case reports and an original research article. This collection encompasses studies spanning fundamental biology, translational neuroscience, innovative disease models, biomarker discovery, and emerging therapeutic strategies. Rather than viewing tumors in isolation, the articles in this issue collectively illustrate that cancers exist within dynamic neurobiological ecosystems in which neuronal activity, glial function, extracellular matrix remodeling, developmental signaling pathways, and immune regulation collectively shape disease progression.

The complementary expertise of the editors reflects this interdisciplinary vision. Sengupta and Pomeranz Krummel have advanced translational neuro-oncology through studies defining therapeutic vulnerabilities in primary and metastatic brain cancers, including the identification of Type-A GABA (GABA-A) receptor signaling as a regulator of *MYC*-amplified medulloblastoma growth and, more recently, GABA-A receptor-mediated change in bioelectricity and autophagy as a strategy to sensitize brain cancers to different treatment modalities (2–4). Neman and colleagues have contributed to understanding how neurodevelopmental pathways, astrocytes, and the brain microenvironment facilitate metastatic colonization of the central nervous system (5) This research illustrates how brain metastases actively exploit neural biology rather than simply invade neural tissue. Building upon this framework, Neman et al. have shown that metastatic and embryonal tumors actively rewire neural and metabolic programs to colonize the central nervous system (6–8).

Several articles in this collection reinforce the concept that reciprocal communication between neurons and tumor cells is a defining hallmark of cancer neuroscience. Feng et al. review how neuronal activity promotes glioma proliferation, invasion, and remodeling of local neural circuits as revealed by examining glioma–neuron interactions and neural plasticity. Complementing this review, Minns et al. provide an insightful analysis how glioma electrophysiology extends this concept by demonstrating that electrical activity itself may serve as both a biomarker of disease progression and a therapeutic target. These reviews reinforce the emerging concept that tumor bioelectricity represents a previously underappreciated dimension of cancer biology, as highlighted in the studies of Sengupta and Pomeranz Krummel.

A recurring theme in several of the articles is the importance of physiologically relevant experimental models. Ishan et al. details how three-dimensional human neural, organoid-based approaches recreate neuron–glia–tumor interactions, more faithfully than conventional culture models, thus providing suitable platforms for study of tumor evolution, therapeutic resistance, and patient-specific treatment responses. Such advances are expected to accelerate translational neuro-oncology by bridging mechanistic discovery and precision medicine.

The collection also highlights the molecular diversity underlying neuro–tumor communication. Li et al. focus on review of NELL2 signaling to illustrate how developmental pathways regulating neuronal differentiation may become dysregulated during tumorigenesis, while Arsentiev et al. examine EMILIN-1 to illustrate that extracellular matrix components can actively shape tumor behavior through regulation of cell signaling and tissue architecture. Together these contributions expand cancer neuroscience beyond synaptic communication alone, demonstrating that developmental biology and extracellular matrix remodeling are equally important determinants of tumor progression.

While a significant focus on neuro-tumor communication has centered on adult primary brain tumors, Chien et al. review what is known regarding the most common pediatric brain tumor, medulloblastoma, and its tumor microenvironment. Medulloblastoma is an embryonal tumor of the cerebellum. Medulloblastomas fall into one of four molecular subgroups, Wingless/Int-1, Sonic-Hedgehog, Group 3, and Group 4. What role the Wingless/Int-1 and Sonic-Hedgehog oncogenic signaling play in the tumor microenvironment and how does GABAergic (Group 3) and Glutamatergic (Group 4) signaling contribute to cancer-brain crosstalk have emerged as fascinating areas of investigation. As highlighted, medulloblastoma may offer a more experimentally tractable cancer model to investigate such phenomena.

An additional strength of this Research Topic is its emphasis on neuroimmune interactions and therapeutic translation. Andrews et al. review neurobiological pathways driving brain tumor progression and discuss how neural signaling influences immune responses within the central nervous system as well as identify opportunities for combining neuroscience with immunotherapy. These concepts are complemented by a clinically oriented study by Dzierma et al. evaluating circulating neurofilament light chain and glial fibrillary acidic protein as biomarkers during cranial radiotherapy. As well as this clinical study, the Research Topic includes two case reports that illustrate both the promise and the current challenges of translating cancer neuroscience into improved patient care. A case report by Qi et al. reports on use of the MET tyrosine kinase inhibitor savolitinib for lung to brain and leptomeningeal metastases. A case report by Hao et al. in this collection illustrates the diagnostic complexity that persists even as neuro-oncology becomes increasingly molecularly driven, describing a cerebellar presentation of ALK-positive anaplastic large cell lymphoma that was initially mistaken for infectious encephalitis and was ultimately treated successfully with the CNS-penetrant ALK inhibitor alectinib once confirmed by biopsy.

It is also worth emphasizing that cancer neuroscience extends well beyond the central nervous system. Tumor–nerve interactions have now been described throughout the peripheral nervous system, where sympathetic, parasympathetic, and sensory fibers regulate tumor initiation, immune evasion, and metastatic spread across a wide range of solid malignancies (9). Within the gastrointestinal tract specifically, the enteric nervous system and its resident glia are increasingly recognized as active participants in tumorigenesis, shaping epithelial proliferation, the local immune microenvironment, and invasion in gastric and colorectal cancer (10). Considered alongside the CNS-focused studies in this collection, this broader literature indicates that reciprocal neuron–tumor signaling is a general principle of cancer biology rather than a phenomenon unique to brain and spinal cord malignancies, and that the field’s future will depend on considering central, peripheral, and enteric neural contributions in parallel.

Collectively, the articles in this issue demonstrate that cancer neuroscience has matured into a translational discipline capable of informing biomarker discovery, therapeutic development, and precision oncology. Advances in single-cell genomics, spatial transcriptomics, advanced imaging, organoid technologies, and computational biology will continue to deepen our understanding of neuro–tumor ecosystems and identify new opportunities for intervention.

We thank all authors, reviewers, and the Frontiers editorial staff for their contributions to this Research Topic. We hope this collection stimulates further interdisciplinary collaboration among neuroscientists, oncologists, immunologists, engineers, and clinician-scientists. By integrating mechanistic biology with translational innovation, the studies presented here establish an important foundation for future discoveries and underscore the promise of cancer neuroscience to improve outcomes for patients with primary and metastatic malignancies.
